# Supplementary material for: Quantifying the gender gap in the HIV care cascade in southern Mozambique: We are missing the men
Source: PLoS One. 2021 Feb 12;16(2):e0245461. doi: 10.1371/journal.pone.0245461 (PMC7880488; doi:10.1371/journal.pone.0245461)
Supplement: S1 Fig — (A) Age-specific and sex-specific proportion of HIV-infected individuals who were aware of their serostatus at the time of the survey; (B) the proportional difference in serostatus awareness between the two sexes. The graph shows the point estimates plus 95% confidence intervals (CI) for the percent difference between female and male awareness of their HIV status. (DOCX) [file pone.0245461.s001.docx]

**S1 Figure. Progress toward the first 90 target in rural southern Mozambique.** (A) Age-specific and sex-specific proportion of HIV-infected individuals who were aware of their serostatus at the time of the survey; (B) the proportional difference in serostatus awareness between the two sexes. The graph shows the point estimates plus 95% confidence intervals (CI) for the percent difference between female and male awareness of their HIV status.

(**A)**

| **Age category** | **Women** | **95%CI** | **Men** | **95%CI** | **p-Value** |
| --- | --- | --- | --- | --- | --- |
| <25 | 84.3% | (78.7-88.8) | 58.6% | (44.9-71.4) | <0.001 |
| 25-34 | 93.2% | (90.9-95.1) | 71.5% | (65.5-77.1) | <0.001 |
| 35-44 | 88.6% | (85.6-91.2) | 83.0% | (77.4-87.7) | 0.035 |
| 45-64 | 86.4% | (82.7-89.5) | 85.2% | (79.1-90.1) | 0.714 |
| >64 | 82.1% | (69.6-91.1) | 66.7% | (47.2-82.7) | 0.108 |
| Total | 88.9% | (87.4-90.4) | 77.1% | (73.9-80.1) | <0.001 |

**(B)**

Gap Women Men (%)

(positive values favor women)
