# Supplementary material for: Quantifying the gender gap in the HIV care cascade in southern Mozambique: We are missing the men
Source: PLoS One. 2021 Feb 12;16(2):e0245461. doi: 10.1371/journal.pone.0245461 (PMC7880488; doi:10.1371/journal.pone.0245461)
Supplement: S2 Fig — Unadjusted cumulative proportion of LTFU after ART initiation over time. The unadjusted sub-distribution hazard ratio (SHR) of LTFU for men vs women was 2.13 (95% CI 1.34 to 3.38). (DOCX) [file pone.0245461.s002.docx]

**S2 Figure. Cumulative incidence of loss to follow-up (LTFU) after antiretroviral therapy (ART) initiation among men and women in rural southern Mozambique.** Unadjusted cumulative proportion of LTFU after ART initiation over time. The unadjusted sub-distribution hazard ratio (HR) of LTFU for men vs women was 2.13 (95% CI 1.34 to 3.38).


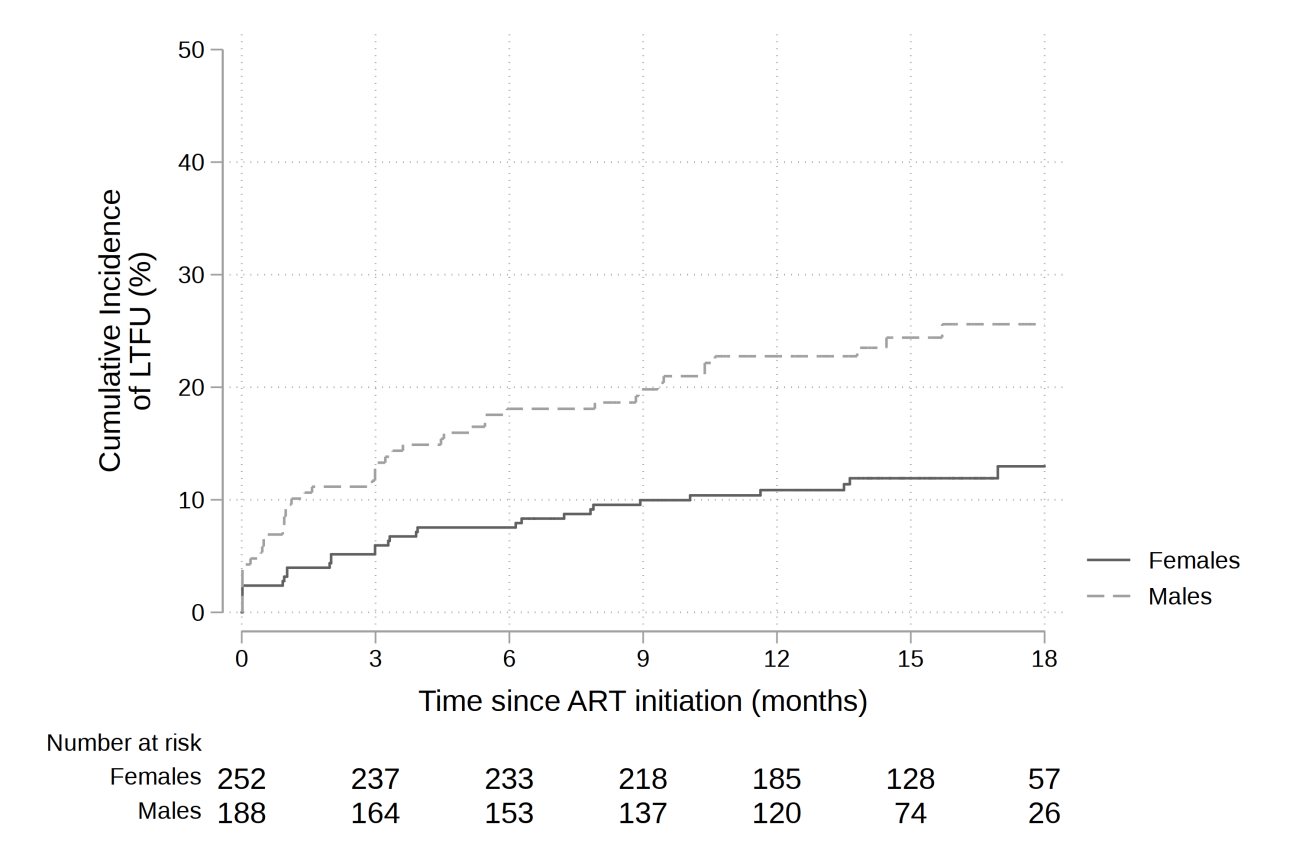


Female

Male

Female

Male
