# Supplementary material for: Quantifying the gender gap in the HIV care cascade in southern Mozambique: We are missing the men
Source: PLoS One. 2021 Feb 12;16(2):e0245461. doi: 10.1371/journal.pone.0245461 (PMC7880488; doi:10.1371/journal.pone.0245461)
Supplement: S1 Table — (DOCX) [file pone.0245461.s003.docx]

**Supporting Information**

**S1 Table. Univariate and multivariable models of factors associated with loss to follow-up at 18 months after initiation of antiretroviral therapy in rural southern Mozambique.**

|  |  | **Loss to follow-up** | | | | | | |
| --- | --- | --- | --- | --- | --- | --- | --- | --- |
|  |  |  | **Univariate** | | | **Multivariable** | | |
|  | **N** | **n (%)** | **SHR** | **95% CI** | **p-value** | **SHR** | **95% CI** | **p-value** |
| **Testing modality** | |  |  |  |  |  |  |  |
| VCT | 174 | 32 (18·4) | Reference |  | 0·252 | Reference |  | 0·336 |
| PICT | 180 | 33 (18·3) | 1·03 | [0·64, 1·67] |  | 0·91 | [0·56, 1·49] |  |
| HBT | 86 | 9 (10·5) | 0·57 | [0·27, 1·18] |  | 0·58 | [0·28, 1·20] |  |
| **Age category (years)** | |  |  |  |  |  |  |  |
| 18–24 | 68 | 8 (11·8) | Reference |  | 0·112 | Reference |  | 0·276 |
| 25–34 | 160 | 33 (20·6) | 1·80 | [0·83, 3·91] |  | 1·40 | [0·61, 3·10] |  |
| 35–44 | 109 | 22 (20·2) | 1·76 | [0·78, 3·96] |  | 1·39 | [0·59, 3·27] |  |
| ≥45 | 103 | 11 (10·7) | 0·89 | [0·36, 2·21] |  | 0·74 | [0·29, 1·90] |  |
| **Sex** |  |  |  |  |  |  |  |  |
| Female | 252 | 30 (11·9) | Reference |  |  | Reference |  |  |
| Male | 188 | 44 (23·4) | 2·13 | [1·34, 3·38] | 0·001 | 1·89 | [1·12, 3·17] | 0·018 |
| **WHO Stage** | |  |  |  |  |  |  |  |
| I–II | 401 | 61 (15·2) | Reference |  | 0·004 | Reference |  |  |
| III–IV | 39 | 13 (33·3) | 2·47 | [1·34, 4·52] |  | 1·87 | [0·98, 3·56] | 0·058 |

Fine and Gray competing risk model analysis estimating determinants of loss to follow-up up to 18 months after initiation of antiretroviral therapy (N=440).

Abbreviations: VCT, voluntary counselling and testing; PICT, provider-initiated counselling and testing; HBT, home-based testing; SHR, sub-distribution hazard ratio; aSHR, adjusted sub-distribution hazard ratio; CI, confidence interval.
