# Supplementary material for: Quantifying the gender gap in the HIV care cascade in southern Mozambique: We are missing the men
Source: PLoS One. 2021 Feb 12;16(2):e0245461. doi: 10.1371/journal.pone.0245461 (PMC7880488; doi:10.1371/journal.pone.0245461)
Supplement: S2 Table — (DOCX) [file pone.0245461.s004.docx]

**S2 Table. Univariate and multivariable models of factors associated with mortality during 24 months after HIV diagnosis in rural southern Mozambique.**

|  |  | **Mortality** | | | | | | |
| --- | --- | --- | --- | --- | --- | --- | --- | --- |
|  |  |  | **Univariate** | | | **Multivariable** | | |
|  | **N** | **n (%)** | **SHR** | **95% CI** | **p-value** | **SHR** | **95% CI** | **p-value** |
| **Sex** |  |  |  |  |  |  |  |  |
| Female | 629 | 35 (5·6) | Reference |  | 0·015 | Reference |  | 0·230 |
| Male | 493 | 46 (9·3) | 1·72 | [1·11, 2·67] |  | 1·32 | [0·84, 2·09] |  |
| **Age category (years)** |  |  |  |  |  |  |  |  |
| 18–24 | 231 | 3 (1·3) |  |  |  |  |  |  |
| 25–34 | 369 | 23 (6·2) |  |  |  |  |  |  |
| 35–44 | 258 | 24 (9·3) |  |  |  |  |  |  |
| ≥45 | 237 | 31 (13·1) |  |  |  |  |  |  |
| Age (years) |  |  | 1·04 | [1·02, 1·05] | <0·001 | 1·04 | [1·03, 1·06] | <0·001 |
| **WHO Stage (N=802)** | |  |  |  |  |  |  |  |
| I–II | 757 | 49 (6·5) | Reference |  | 0·013 | Reference |  | 0·002 |
| III–IV | 45 | 9 (20·0) | 3·42 | [1·68, 6·96] |  | 3·00 | [1·41, 6·41] |  |
| Unknown | 320 | 23 (7·2) | 1·12 | [0·68, 1·83] |  | 0·50 | [0·24, 1·02] |  |
| **Advanced disease** CD4 <100 cells/ mm3 **(N=699)** | | |  |  |  |  |  |  |
| No | 592 | 27 (4·6) | Reference |  | <0·001 | Reference |  | <0·001 |
| Yes | 107 | 19 (17·8) | 4·29 | [2·38, 7·71] |  | 4·21 | [2·24, 7·91] |  |
| Unknown | 423 | 35 (8·3) | 1·86 | [1·12, 3·07] |  | 2·12 | [0·96, 4·67] |  |
| **In ART** |  |  |  |  |  |  |  |  |
| No | 606 | 49 (8·1) | Reference |  | 0·221 | Reference |  | 0·026 |
| Yes | 516 | 32 (6·2) | 0·76 | [0·49, 1·18] |  | 0·46 | [0·23, 0·91] |  |

Fine and Gray competing risk model analysis estimating determinants of mortality up to 24 months after initiation of antiretroviral therapy.

Univariate model includes N=1122 and 81 failures.

Abbreviations: ART, antiretroviral therapy; SHR, sub-distribution hazard ratio; aSHR, adjusted sub-distribution hazard ratio; CI, confidence interval
